# Prediction and mediation analysis for treatment responses to combined cognitive and physical training for older adults

**DOI:** 10.1038/s41598-024-61407-6

**Published:** 2024-05-08

**Authors:** I.-Ching Chuang, I.-Chen Chen, Yih-Ru Wu, Kuan-Yi Li

**Affiliations:** 1 Department of Occupational Therapy and Graduate Institute of Behavioral Sciences, College of Medicine, Chang Gung University, No. 259, Wunhua 1st Rd., Gueishan Township, Taoyuan, 333 Taiwan; 2 Department of Neurology, Chang Gung Memorial Hospital at Linkou, Taoyuan, Taiwan; 3 Department of Occupational Therapy, College of Nursing and Health Sciences, Da-Yeh University, Changhua, Taiwan; 4 College of Medicine, Chang Gung University, Taoyuan, Taiwan; 5 Healthy Aging Research Center, Chang Gung University, Taoyuan, Taiwan

**Keywords:** Combined cognitive and physical training, Processing speed, Functional mobility, Instrumental activities of daily living, Predictor, Mediator, Diseases, Health care

## Abstract

Diminished cognitive and physical functions negatively affect the daily functions of individuals. Although combined cognitive and physical training prevents instrumental activities of daily living (IADL) disability in older adults, no predictive model or mediation analysis of IADL after combined training exists. This study aims to employ prediction and mediation analysis to identify the predictors of IADL performance and to elucidate the mediators of the association between baseline global cognition and subsequent IADL performance following combined cognitive and physical training. This study involved 177 participants aged 60 years and older who underwent combined training. Cognitive function was measured with the Montreal Cognitive Assessment (MoCA), Digit Symbol Substitution Test (DSST), Color Trails Test, Word List, and a dual task; physical function with the Timed Up and Go (TUG) test; daily function with the Lawton IADL Scale. We conducted regression analyses to identify the predictors of IADL performance, and mediation analysis to examine whether DSST and TUG mediate the relationship between MoCA and IADL. The pre-intervention DSST and TUG were significant independent predictors of post-intervention IADL. The association between the pre-intervention MoCA and post-intervention IADL was mediated by pre-intervention DSST and TUG. This study highlighted the importance of measuring and improving processing speed and functional mobility before and during interventions to enhance IADL outcomes.

Trial registration: NCT03619577, 23/07/2018; NCT04689776, 29/12/2020.

## Introduction

Cognitive decline, such as deterioration in attention, processing speed, memory, and executive functions^1–3^ and physical decline, such as reduced muscle mass, weakness, and slowness^2,4–6^, commonly occur with increasing age. Diminished cognitive and physical functions negatively affect individuals’ abilities to perform everyday activities, such as managing finances and preparing meals (i.e., instrumental activities of daily living, IADL) and contribute to dependency in daily life, which increases the care burden and costs for families and society. Previous studies have shown that combined cognitive and physical training prevents disability in IADL and is beneficial for the ability to perform IADL in older adults^7–9^. Predictive modeling and mediation analysis of IADL performance after combined training can provide valuable information for clinical decision-making and outcome research; however, but this issue has not been widely addressed.

The ability to perform IADL is an essential cornerstone of independent living and personal autonomy. Previous studies have shown that IADL ability depends on cognitive^10,11^ and physical functions^12–14^. Existing evidence has shown that multi-dimensional cognitive abilities are associated with IADL in older adults^15–23^; these abilities include processing speed (the ability to process information rapidly)^24^, sustained attention (the ability to maintain vigilance on a particular task over an extended period)^25,26^, verbal memory (the ability to store and recall phonological information)^27^, and executive functions (a collection of top-down mental control processes, including inhibition, working memory, and cognitive flexibility)^28^. Similarly, global cognition, referring to a family of cognitive capacities, including orientation, attention, memory, language, and executive function^29^ is related to functional independence^30–32^. On the other hand, previous studies show that functional mobility, referring to the ability to independently and safely move across environments to engage in functional activities^33,34^, is related to IADL performance in older adults^13,35,36^. These studies highlight the cognitive and physical abilities concurrently in relation to the IADL performance in this population. However, existing evidence does not suggest which abilities of older adults could serve as indicators to predict IADL outcomes following combined training. Establishing predictive modeling for IADL outcomes after combined cognitive and physical training can identify the characteristics of individuals who would benefit the most, which helps clinical practitioners recruit a certain population and effectively deliver tailored interventions.

Since age-related deterioration in cognition that may impede functional independence is a primary concern, combined cognitive and physical training is intended to enhance cognition in older adults based on the guided plasticity facilitation framework^37^. Previous studies have demonstrated that combined training improves the global cognition of older adults^38–42^, and that global cognition and IADL performance show a strong association^30–32^. However, given global cognition as a collection of cognitive capacities, it remains unclear how global cognition may improve IADL performance following combined training. Numerous variables may play a role in the relationship between global cognition and IADL performance. Evidence suggests that in addition to global cognition, improved processing speed and functional mobility exert a positive impact on the IADL performance of older adults^13,19,35,36,43,44^. Processing speed is the fundamental ability to rapidly process information and lay the basis for higher-order cognitive processes^24,45^. Functional mobility is the ability to move across environments and quickly switch motor programs according to changes in the environment and task requirements^33,46^, which involves both physical and cognitive functions. The mechanism underlying the association between global cognition and IADL performance needs to be elucidated, which would be helpful in targeting specific abilities for interventions.

Developing interventions to prevent disability in IADL among older adults is crucial, and a better understanding of the factors that predict the response to combined cognitive and physical training is needed to direct interventions to those who will potentially benefit from it. Through a mediation analysis, we gain insight and acquire a deep understanding of the pathways of intervention effects, and this information helps to determine efficient and efficacious intervention strategies. We investigated whether better global cognition before combined training would result in better processing speed and functional mobility and, in turn, contribute to better IADL performance after combined training. The aims of this study were to (1) identify the characteristics of older adults who would benefit most in terms of IADL performance from combined cognitive and physical training, and (2) clarify the contribution of processing speed and functional mobility to the association between baseline global cognition and subsequent IADL performance of older adults after combined training.

## Materials and methods

### Participants

This study was a secondary analysis of data from two pre-post studies of combined cognitive and physical training with convenience sampling. Those who were (1) aged 60 years or over, (2) having the ability to follow instructions (a score of Mini-Mental State Evaluation ≥ 20), (3) not diagnosed with dementia by physicians, and (4) having no difficulties in basic ADLs were recruited in this study. Individuals with self-reported diagnoses of neurological disorders or unstable medical conditions were excluded.

### Procedure

The study was conducted in accordance with the Declaration of Helsinki and approved by the Research Ethics Committee of National Taiwan University (201711EM006, 201912EM016) for studies involving humans. Participants were invited from community facilities or daycare centers. All participants were screened to ensure eligibility in the study. According to the temporal order of cognitive and physical training, the combined interventions can be categorized into sequential (at a separate time) and simultaneous (at the same time) training. The participants engaged in either a sequential or simultaneous combination of cognitive and physical training. The instructors were community occupational therapists. Before conducting the interventions, all instructors were trained with the same program regarding motor-cognitive training concepts to minimize staff training weaknesses due to multisite sampling. Four researchers, who were unaware of the assigned interventions, evaluated the participants’ daily cognitive and physical functions before and after the interventions. If the participants were absent from more than 30% of the sessions, they were excluded from the study.

### Intervention

In the present study, we conducted sequential and simultaneous cognitive and physical training. The doses of the two interventions were matched at 120 min per session, once a week, for 12 sessions. Each session consisted of physical and cognitive exercises. The cognitive components included attention, processing speed, short-term memory, working memory, visuospatial skills, calculation, and language; one or more cognitive components were included in every session. The physical components consisted of stretching, muscle strengthening, aerobic, and balance exercises.

### Measures

For prediction analysis, the potential predictors were global cognition, processing speed, sustained attention, verbal memory, executive functions, and functional mobility, which were assessed using the Montreal Cognitive Assessment (MoCA), Digit Symbol Substitution Test (DSST), Color Trails Test (CTT), Word List (WL), a dual task that we designed, and Timed Up and Go (TUG) test, respectively. For mediation analysis, the mediator variables were processing speed and functional mobility, which were assessed using the DSST and TUG, respectively. For both prediction and mediation analyses, the outcome variable was IADL performance, which was assessed using the Lawton IADL Scale.

### MoCA

The MoCA is a validated and sensitive tool for evaluating general cognition and detecting mild cognitive impairment. It is also widely recognized as a reliable measure of cognitive function^47^. It comprises 12 items that assess the executive functions, visuospatial abilities, language, short-term memory, attention, concentration, working memory, and orientation to time and place. The education level of the examinee was considered; one point was added to the total score if an examinee had 12 years or less of formal education. The scores on the MoCA ranged from 0 to 30, with higher scores indicating a higher function. A total score of 26 or above is considered normal and a total score of 19 to 25 indicates mild cognitive impairment^29^. In this study, we used a certified Chinese paper version (MoCA-T), this measurement tool has good reliability and validity^48^.

### DSST

The DSST is designed to assess the processing speed; it is a subtest from the corpus of the Chinese version of the Wechsler Adult Intelligence Scale, Third Edition^49^. A participant is asked to transcribe a unique geometric symbol with its corresponding Arabic number. Initially, the participant was presented with a series of boxes containing numbers from 1 to 9 sequentially; there was a corresponding geometric symbol under each number. Subsequently, he/she is shown a series of boxes containing numbers in the top boxes and blank boxes below them, and is asked to copy the corresponding geometric symbol under each number. After the practice trial, the participant copied as many geometric symbols as possible in 120 s. The raw score was the number of correct items and was transformed into a scaled score based on the age range. It is considered to be a valid and sensitive measure of cognitive dysfunction, particularly in the working memory^50^.

### CTT

The CTT was designed to assess sustained attention. Each participant was presented with numbered circles printed on pink and yellow backgrounds. For CTT Part 1 (CTT1), the participant was asked to connect circles numbered 1–25 in sequence using a pencil, as rapidly as possible. For CTT Part 2 (CTT2), the participant was asked to rapidly connect circles numbered 1–25 in sequence, but alternated between pink and yellow. The time taken to complete each trial, errors, near-misses, and prompts were recorded. The time was transformed into scaled time based on sex, age, and years of formal education. In this study, we used the Taiwanese Version of CTT^51^. This test has shown good reliability and validity in healthy adults^51^.

### WL

WL is designed to assess the ability to recall information presented in verbal form immediately after a 30-min delay. It is a subtest from the corpus of the Chinese version of the Wechsler Memory Scale, Third Edition^52^. For WL Part I (WL-I), a participant is verbally presented with a 12-item list of unrelated words over a series of four trials and then is asked to recall the list immediately. For WL Part II (WL-II), 30 min after WL-I, the participants were asked to recall the 12-item list of words verbally presented earlier. The raw score of WL-I is the number of the sum of the correct responses of the four trials and that of WL-II is the number of the correct responses of one trial. Raw scores were transformed into scaled scores based on the age range.

### Dual task: Box and Block Test and Serial Seven Test

We designed this dual task to assess executive functions. Each participant was instructed to simultaneously conduct the Box and Block Test and Serial Seven Test (SST) for 60 s. Each participant was seated at a table and presented with a rectangular box divided into two square compartments. One hundred and fifty colored wooden blocks were placed in each compartment. A participant is asked to move a block, one at a time, from one compartment to the other as many as he/she can for 60 s^53^. At the same time, a participant is asked to conduct serial subtraction by seven beginning with 300. The number of blocks moved was recorded. The difference between the correct and incorrect SST responses was calculated as the corrected number.

### TUG

The TUG is a reliable and valid test designed to assess the mobility, balance (static and dynamic), and walking ability. The participants were asked to perform sequential motor tasks: stand up from a chair, walk a distance of 3 m, turn around, walk back to the chair, and sit down. Timing begins at the verbal instruction “go” and stops when a participant is seated. The score given is the time taken to complete the test, in seconds^54^. In this study, the score is calculated as the average time of three trials for each participant, in seconds.

### Lawton IADL scale

The Lawton IADL Scale is a valid tool designed to evaluate the ability to perform tasks and detect early functional decline. The reliability and validity of the Lawton IADL scale have been established. In a semi-structured interview lasting 10 to 15 min, a participant was asked to describe in detail how IADLs were currently being performed. The IADL include eight items: using the telephone, shopping, preparing food, doing laundry, housekeeping, using transportation, handling medications, and managing finances. Each item was rated based on the level of competence, from independent in performing the activity to not performing it at all. Higher scores indicate better functioning^55^.

### Data analysis

Descriptive statistics were used to present the demographic characteristics of participants. Paired *t*-tests were used to compare pre- and post-intervention differences. Pearson’s correlation analysis was used to analyze the demographic variables and pre-intervention functional measures that were potentially related to post-intervention IADL. A Pearson’s *r-*value of 0.35 was considered the cut-off value in this study. Regression analyses were conducted to identify significant predictors of post-intervention IADL. A mediation analysis was used to clarify the mechanism underlying the observed relationship between an independent and dependent variable that could be explained by a third variable known as a mediator variable. We conducted a parallel mediation analysis to examine whether pre-intervention MoCA influenced post-intervention IADL through mediator variables, pre-intervention DSST, or TUG. We conducted serial mediation analyses to examine the direct and indirect effects of pre-intervention MoCA on post-intervention IADL while modeling a process in which better pre-intervention MoCA caused better pre-intervention DSST, and, in turn, caused better pre-intervention TUG, and, so forth, concluding with better post-intervention IADL as the final consequence^35^. Mediation analyses were conducted with age, sex, group, years of formal education, and pre-intervention IADL as covariates.

Data analysis for this study was performed using SAS software, Version 9.4 of the SAS System. Mediation analyses were performed using the PROCESS functions for SAS designed by Hayes (2018). Specifically, we employed PROCESS Model 4 for the parallel mediation model and Model 6 for the serial mediation model. All mediation models were analyzed using 5000 bootstrap samples and 95% bias-corrected confidence intervals (CIs).

### Ethics approval and consent to participate

This study was conducted in accordance with the Declaration of Helsinki and approved by the Research Ethics Committee of National Taiwan University (201711EM006, 201912EM016) for studies involving humans. Before conducting the study, all participants were provided with a written and oral explanation of the study protocol. After obtaining written informed consent, the participants started to participate in the study. As this article does not include the identifiable data of any participant in any form, consent for publication is not applicable.

## Results

### Demographic characteristics

Initially, 177 participants were recruited for the study. However, participants who were absent for more than 30% of the intervention sessions were excluded from the data analysis, and twelve and seven participants in the sequential and simultaneous groups, respectively were excluded. Therefore, 158 participants completed the pre- and post-intervention measures and were included in the data analysis. The demographic characteristics and pre- and post-intervention measures are presented in Table 1. The participants showed significant improvements in the IADL, MoCA, DSST, WL-I, and WL-II scores after the intervention.

**Table 1:** Characteristics and functional measures of all participants.

	Baseline/Pre-intervention		Post-intervention		*t* (157)	*p*
	*M* (*SD*) or	*N* (%)	*M* (*SD*) or	*N* (%)		
Age (yr)	73.73	(7.28)	–			
Sex						
Female	109	(68.99)	–			
Male	49	(31.01)	–			
Education (yr)	8.97	(3.93)	–			
Group						
Simultaneous	56	(35.44)	–			
Sequential	102	(65.56)	–			
MMSE	26.44	(3.14)	26.49	(3.21)	0.025	0.402
IADL	28.91	(3.62)	29.26	(3.22)	1.75	0.041
MoCA	23.25	(4.91)	23.66	(5.02)	1.96	0.026
DSST	10.21	(2.67)	10.63	(2.95)	3.85	< 0.001
WL-I	9.71	(3.35)	10.75	(3.98)	5.28	< 0.001
WL-II	10.91	(3.01)	11.22	(3.33)	1.74	0.042
CTT1 (s)	53.82	(36.61)	58.53	(59.28)	0.62	0.732
CTT2 (s)	127.11	(75.03)	121.11	(80.30)	1.35	0.910
SST (No.)	5.65	(4.14)	5.73	(4.36)	0.97	0.167
TUG (s)	11.84	(5.04)	11.31	(3.63)	1.29	0.994

Education, years of formal education; SST, the number of correctly answering serial subtraction by seven in a dual task.

*CTT1* Color Trails Test Part 1, *CTT2* Color Trails Test Part 2, *DSST* Digit Symbol Substitution Test, *IADL* Lawton Instrumental Activity of Daily Living, *M* mean, *MMSE* Mini-Mental State Evaluation, *MoCA* Montreal of Cognitive Assessment, *SD* standard deviation, *SST* Serial Seven Test, *TUG* Timed Up and Go, *WL-I* Word List-Part I, *WL-II* Word List-Part II.

### Correlation analyses

We examined the correlations between age, years of formal education, pre-intervention IADL, MoCA, DSST, WL-I, WL-II, CTT1, CTT2, dual-task, TUG, and post-intervention IADL; the results are presented in Table 2.

**Table 2:** Pearson correlation coefficients (*r*) among variables.

		1	2	3	4	5	6	7	8	9	10	11
1	IADL_Post_											
2	Age	− 0.365^‡^										
3	Edu	0.07	− 0.098									
4	IADL_Pre_	0.729^‡^	− 0.445^‡^	− 0.002								
5	MoCA_Pre_	0.530^‡^	− 0.438^‡^	0.250^†^	0.557^‡^							
6	DSST_Pre_	0.419^‡^	− 0.204*	0.389^‡^	0.400^‡^	0.628^‡^						
7	WL-I_Pre_	0.306^‡^	− 0.291^‡^	0.256^†^	0.439^‡^	0.721^‡^	0.650^‡^					
8	WL-II_Pre_	0.323^‡^	− 0.170*	0.211^†^	0.376^‡^	0.607^‡^	0.510^‡^	0.756^‡^				
9	CTT1_Pre_	− 0.193	0.065	0.169	− 0.402^‡^	− 0.444^‡^	− 0.446^‡^	− 0.288^†^	− 0.225*			
10	CTT2_Pre_	− 0.274^†^	0.137	0.088	− 0.416^‡^	− 0.505^‡^	− 0.548^‡^	− 0.455^‡^	− 0.374^‡^	0.732^‡^		
11	SST_Pre_	0.305^†^	− 0.263^†^	0.318^†^	0.265^†^	0.613^‡^	0.548^‡^	0.636^‡^	0.568^‡^	− 0.200	− 0.345^‡^	
12	TUG_Pre_	− 0.577^‡^	0.513^‡^	− 0.151	− 0.489^‡^	− 0.555^‡^	− 0.220*	− 0.329^‡^	− 0.213*	0.235*	0.227*	− 0.299^†^

*p < 0.05, ^†^*p* < 0.01, ^‡^*p* < 0.001. *Edu* years of formal education, *SST* the number of correctly answering serial subtraction by seven in a dual task, *CTT1* Color Trails Test Part 1, *CTT2* Color Trails Test Part 2, *DSST* Digit Symbol Substitution Test, *IADL* Lawton Instrumental Activity of Daily Living, *MoCA* Montreal of Cognitive Assessment, *pre* pre-intervention, *post* post-intervention, *SST* Serial Seven Test, *TUG* Timed Up and Go, *WL-I* Word List-Part I, *WL-II* Word List-Part II.

### Univariate and multivariate regression analyses

First, we conducted univariate regression analyses to identify the potential predictors of post-intervention IADL. The results showed that age, pre-intervention IADL, MoCA, DSST, and TUG were five significant predictors (Table 3). Subsequently, we included age, pre-intervention MoCA, DSST, and TUG as predictors for multivariate regression analysis while controlling for pre-intervention IADL, sex, and group. The results showed that pre-intervention DSST and TUG significantly predicted post-intervention IADL, with an adjusted *R*^2^ of 0.514 (Table 3).

**Table 3:** Predictors of post-intervention IADL.

	Univariate			Multivariate				
	Std β	*SE*	*t*	Std β	*SE*	*t*	VIF	Adj *R*^2^
Age	− 0.3651	0.0325	− 4.88^‡^	0.1053	0.0263	1.28	1.60	0.514
Sex	− 0.1523	0.5489	− 1.92	− 0.0334	0.3334	− 0.48	1.13	
Group	− 0.0885	0.5604	− 1.12	0.0230	0.2938	0.34	1.07	
IADL_Pre_	0.7286	0.0488	13.29^‡^	0.4740	0.0684	6.06^‡^	1.45	
MoCA_Pre_	0.5302	0.0445	7.81^‡^	0.0702	0.0484	0.73	2.18	
DSST_Pre_	0.4188	0.0822	5.70^‡^	0.1586	0.0658	2.00*	1.49	
TUG_Pre_	− 0.5769	0.0343	− 7.61^‡^	− 0.3111	0.0371	− 3.69^‡^	1.69	

*p < 0.05, ^†^*p* < 0.01, ^‡^*p* < 0.001.

*Adj* adjusted, *DSST* Digit Symbol Substitution Test, *IADL* Lawton Instrumental Activity of Daily Living, *MoCA* Montreal of Cognitive Assessment, *pre* pre-intervention, *post* post-intervention, *SE* standard error, *Std* standardized, *TUG* Timed Up and Go, *VIF* variance inflation factor.

### Parallel mediation analysis

**Figure 1:**
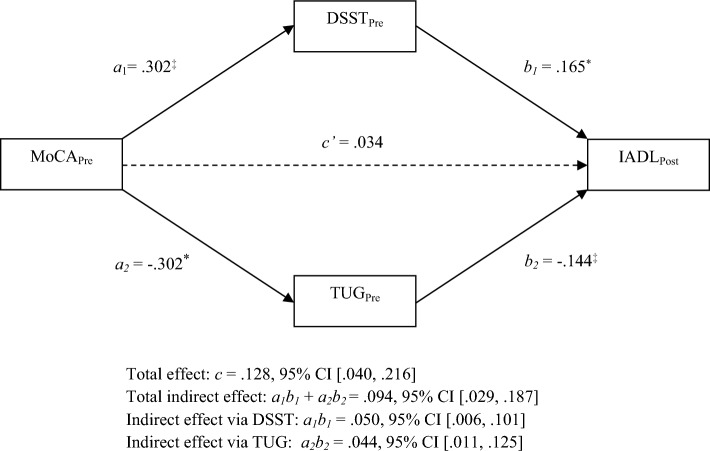
Parallel mediation model. Indirect effects of pre-intervention MoCA on post-intervention IADL through pre-intervention DSST and TUG. The direct effect was insignificant depicted by dotted line. *p < 0.05, ^†^*p* < 0.01, ^‡^*p* < 0.001.

We assumed that the pre-intervention DSST and TUG mediated the relationship between the pre-intervention MoCA and post-intervention IADL (Fig. 1).

The results showed that better pre-intervention global cognition was associated with higher levels of post-intervention IADL performance (β = 0.128, 95% CI [0.040, 0.216]). Better pre-intervention global cognition was associated with better pre-intervention processing speed (β = 0.302, 95% CI [0.192, 0.413]) and functional mobility (β = − 0.302, 95% CI [− 0.513, − 0.093]). The pre-intervention processing speed and functional mobility were linked to improved post-intervention IADL performance. The total indirect link between pre-intervention global cognition and post-intervention IADL performance via processing speed and functional mobility was significant (β = 0.094, 95% CI [0.029, 0.187]). The direct link between pre-intervention global cognition and post-intervention IADL performance was insignificant after adjusting for the mediators (β = 0.034, 95% CI [− 0.061, 0.130]), supporting a full mediation model (Table 4).

**Table 4:** Mediation models.

Paths	β (SE)	95% CIs	*p*
Parallel mediation model			
**MoCA**_**Pre**_** → IADL**_**Post**_** (total effect: path c)**	0.128 (0.044)	[0.040, 0.216]	0.005
**MoCA**_**Pre**_** → DSST**_**Pre**_** (path a**_**1**_**)**	0.302 (0.056)	[0.192, 0.413]	< 0.001
**DSST**_**Pre**_** → IADL**_**Post**_** (path b**_**1**_**)**	0.165 (0.071)	[0.025, 0.306]	0.021
**MoCA**_**Pre**_** → TUG**_**Pre**_** (path a**_**2**_**)**	− 0.302 (0.106)	[− 0.513, − 0.093]	0.029
**TUG**_**Pre**_** → IADL (path b**_**2**_**)**	− 0.144 (0.038)	[− 0.218, − 0.070]	< 0.001
**Total indirect effect: path a**_**1**_**b**_**1**_** + a**_**2**_**b**_**2**_	0.094 (0.040)	[0.029, 0.187]	–
**MoCA**_**Pre**_** → DSST**_**Pre**_** → IADL**_**Post**_** (indirect effect: path a**_**1**_**b**_**1**_**)**	0.050 (0.024)	[0.006, 0.101]	–
**MoCA**_**Pre**_** → TUG**_**Pre**_** → IADL**_**Post**_** (indirect effect: path a**_**2**_**b**_**2**_**)**	0.044 (0.029)	[0.011, 0.125]	–
MoCA_Pre_ → IADL_Post_ (direct effect: path c’)	0.034 (0.048)	[− 0.061, 0.130]	0.479
Serial mediation models			
**MoCA**_**Pre**_** → IADL**_**Post**_** (total effect: path c)**	0.128 (0.044)	[0.040, 0.216]	0.005
**MoCA**_**Pre**_** → DSST**_**Pre**_** (path a**_**1**_**)**	0.302 (0.056)	[0.192, 0.413]	< 0.001
**DSST**_**Pre**_** → IADL**_**Post**_** (path b**_**1**_**)**	0.165 (0.071)	[0.025, 0.306]	0.021
**MoCA**_**Pre**_** → TUG**_**Pre**_** (path a**_**2**_**)**	− 0.363 (0.119)	[− 0.599, − 0.127]	0.003
**TUG**_**Pre**_** → IADL**_**Post**_** (path b**_**2**_**)**	− 0.144 (0.038)	[− 0.218, − 0.070]	< 0.001
**DSST**_**Pre**_** → TUG**_**Pre**_** (path d**_**21**_**)**	0.198 (0.181)	[− 0.161, 0.557]	0.277
Total indirect effect: path a_1_b_1_ + a_2_b_2_ + a_1_d_21_b_2_	0.092 (0.041)	[0.027, 0.187]	–
**MoCA**_**Pre**_** → DSST**_**Pre**_** → IADL**_**Post**_** (indirect effect: path a**_**1**_**b**_**1**_**)**	0.050 (0.024)	[0.006, 0.101]	–
**MoCA**_**Pre**_** → TUG**_**Pre**_** → IADL**_**Post**_** (indirect effect: path a**_**2**_**b**_**2**_**)**	0.055 (0.030)	[0.013, 0.126]	–
MoCA_Pre_ → DSST_Pre_ → TUG_Pre_ → IADL_Post_ (indirect effect: path a_1_d_21_b_2_)	− 0.011 (0.010)	[− 0.026, 0.017]	–
MoCA_Pre_ → IADL_Post_ (direct effect: path c′)	0.034 (0.048)	[− 0.061, 0.130]	0.479

Paths in bold indicate statistically significant effects.

*CI* confidence interval, *DSST* Digit Symbol Substitution Test, *IADL* Lawton Instrumental Activity of Daily Living, *MoCA* Montreal of Cognitive Assessment, *pre* pre-intervention, *post* post-intervention, *SE* standard error, *TUG* Timed Up and Go.

### Serial mediation analysis

**Figure 2:**
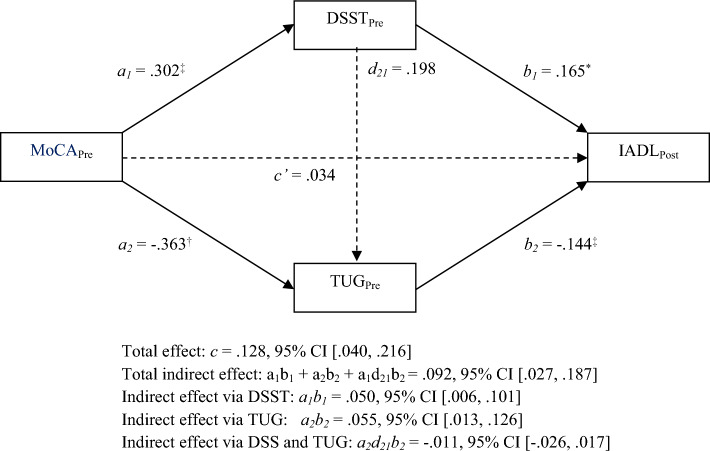
Serial mediation model. Indirect effects of pre-intervention MoCA on post-intervention IADL through pre-intervention DSST and TUG. The direct and indirect effects were insignificant depicted by dotted line. *p < 0.05, ^†^*p* < 0.01, ^‡^*p* < 0.001.

We assumed that increased cognitive function would lead to elevated physical function and enhanced IADL (Fig. 2). However, the results showed that there was no serial causal relationship between the two mediators (Table 4).

## Discussion

To the best of our knowledge, this study is the first to identify the characteristics of older adults who would benefit most in terms of IADL performance after combined cognitive and physical training, and to disentangle the mechanisms underlying the relationship between baseline global cognition and IADL performance following training. The findings showed that the important predictors and mediators of IADL performance in older adults after the intervention were processing speed and functional mobility.

In the univariate regression model, age and global cognition before the intervention were predictors of IADL performance after the intervention, but were no longer predictors in the multivariate regression model. On the other hand, the processing speed and functional mobility before intervention were independent predictors of IADL performance after intervention in both univariate and multivariate regression models. The findings indicated that processing speed and functional mobility before intervention better predicted the ability to perform IADL after intervention than age and global cognition, and that older adults who possessed better baseline performance on processing speed and functional mobility benefited the IADL performance from combined training. Therefore, considering the improvement in IADL performance in the targeted population, evidence suggests that clinical practitioners can measure the processing speed and functional mobility of older adults to recruit participants for combined cognitive and physical training instead of global cognition.

The results of the parallel mediation analysis showed that the processing speed and functional mobility before the intervention fully mediated the relationship between baseline global cognition and IADL performance following intervention in older adults. The current study provides evidence suggesting that global cognition before the intervention by itself may have a small positive effect on the IADL performance after the intervention. However, global cognition can indirectly benefit the IADL performance in older adults by enhancing the processing speed and functional mobility.

Our findings indicate that a high level of global cognition in older adults can boost the processing speed and functional mobility and, in turn, improve IADL performance following the intervention. Processing speed is considered a fundamental component of cognitive ability and is often considered the baseline for higher-order cognitive processes such as memory and executive function^24,45^. Age-related reduction in processing speed may lead to compromised cognitive performance since the cognitive operations that are relevant to cognitive tasks cannot be completed within a limited time and the cognitive information is not simultaneously available for higher-order cognitive processes^1,3,24,45,56–58^. Therefore, an individual requires good processing speed, which supports them to perform complex activities involving higher-order cognition, such as IADL^15,17,21–23^. Our finding coincided with previous studies stating that enhanced processing speed may contribute to lesser limitations in IADL in older adults^59–61^. Therefore, targeting processing speed ability through trainings may be an important priority for promoting healthy aging and maintaining functional independence in older adults, regardless of their baseline global cognitive function. Alternatively, functional mobility requires not only physical but also cognitive function; it involves postural control, balance, locomotion, lower-extremity strength, continuous evaluation of cues from environments, and rapid shifting motor programs based on task demands and changes in environments^33,46^. A prior study also suggested that among older adults, routine walking was a task involving cognitive abilities rather than an automated and rhythmic motor task^62^. Our findings were in line with previous studies that showed that functional mobility was associated with global cognition^63–67^. An individual with good functional mobility is able to go to different places and participate in functional and meaningful activities, such as IADL^13,35,36^. The results align with previous research which determined that elevated functional mobility may lead to the increased ability to perform IADL in older adults^35,36,60,68^. To ameliorate the IADL performance of older adults using an effective intervention strategy, the evidence suggests that the combined cognitive and physical training should target to address and enhance the processing speed and functional mobility of older adults.

### Limitations

The current study had several limitations. First, the convenience sample mainly consisted of women (69.77%) and was area-specific. To increase the generalizability of our findings, a robust sampling strategy and large samples are needed in future research. Second, this was a secondary data analysis study, and we collected data from community-dwelling older adults who participated in either sequential or simultaneous interventions. However, the sample sizes of the two intervention groups were unequal due to practical considerations, and this group was included as a covariate in the analyses. Third, the measurements of the predictors and mediators (DSST and TUG) were performance-based, whereas the measurement of outcomes (IADL) was based on self-reporting. The performance-based measurement of IADL may be considered in future research.

## Conclusions

With a rapidly growing aging population, identifying modifiable factors that can prevent the loss of independence is an urgent priority. To enhance the IADL performance of older adults using effective intervention strategies, our findings provide evidence that clinical practitioners can measure the processing speed and functional mobility of older adults to determine recruitment for combined cognitive and physical training and that the content of combined cognitive and physical training for older adults should aim to improve the processing speed and functional mobility for the best IADL outcomes.

## Data Availability

The datasets used and/or analyzed in the current study are available from the corresponding author upon reasonable request.

## Abbreviations

CTT: Color Trails Test
CTT1: Color Trails Test Part 1
CTT2: Color Trails Test Part 2
DSST: Digit Symbol Substitution Test
IADL: Instrumental Activities of Daily Living
MoCA: Montreal Cognitive Assessment
TUG: Timed Up and Go
WL: Word List
WL-I: Word List-Part I
WL-II: Word List-Part II

## References

[1] Harada CN, Natelson LMC, Triebel KL (2013). Normal cognitive aging. Clin. Geriatr. Med..

[2] Lord SR, Delbaere K, Sturnieks DL (2018). Aging. Handb. Clin. Neurol..

[3] Murman DL (2015). The impact of age on cognition. Semin. Hear..

[4] Billot M (2020). Preserving mobility in older adults with physical frailty and sarcopenia: Opportunities, challenges, and recommendations for physical activity interventions. Clin. Interv. Aging.

[5] Cruz-Jimenez M (2017). Normal changes in gait and mobility problems in the elderly. Phys. Med. Rehabil. Clin. N. Am..

[6] Zullo A (2020). Structural and functional changes in the coupling of fascial tissue, skeletal muscle, and nerves during aging. Front. Physiol..

[7] Chuang IC (2022). Baseline global cognitive function affects cognitive and functional outcomes of combined physical and cognitive training among older adults with cognitive decline. Am. J. Occup. Ther..

[8] Law LLF, Mok VCT, Yau MMK (2019). Effects of functional tasks exercise on cognitive functions of older adults with mild cognitive impairment: A randomized controlled pilot trial. Alzheimer’s Res. Ther..

[9] Fiatarone Singh MA (2014). The Study of Mental and Resistance Training (SMART) study-resistance training and/or cognitive training in mild cognitive impairment: A randomized, double-blind, double-sham controlled trial. J. Am. Med. Dir. Assoc..

[10] Bruderer-Hofstetter M (2020). Development of a model on factors affecting instrumental activities of daily living in people with mild cognitive impairment—A Delphi study. BMC Neurol..

[11] Mlinac ME, Feng MC (2016). Assessment of activities of daily living, self-care, and independence. Arch. Clin. Neuropsychol..

[12] Albert SM, Bear-Lehman J, Anderson SJ (2015). Declines in mobility and changes in performance in the instrumental activities of daily living among mildly disabled community-dwelling older adults. J. Gerontol. Ser. A Biol. Sci. Med. Sci..

[13] Wang DXM (2020). Muscle mass, strength, and physical performance predicting activities of daily living: A meta-analysis. J. Cachexia Sarcopenia Muscle.

[14] Moriyama N (2021). Association of instrumental activities of daily living, physical function and mental health among older returnees after the Fukushima Daiichi nuclear power station accident. Int. J. Environ. Res. Public Health.

[15] Cornelis E (2019). The relationship between basic, instrumental, and advanced activities of daily living and executive functioning in geriatric patients with neurocognitive disorders. Int. J. Geriatr. Psychiatry.

[16] Reppermund S (2011). The relationship of neuropsychological function to instrumental activities of daily living in mild cognitive impairment. Int. J. Geriatr. Psychiatry.

[17] Tomaszewski Farias S (2009). Longitudinal changes in memory and executive functioning are associated with longitudinal change in instrumental activities of daily living in older adults. Clin. Neuropsychol..

[18] Tomaszewski Farias S (2018). Self-perceived difficulties in everyday function precede cognitive decline among older adults in the ACTIVE study HHS Public Access. J. Int. Neuropsychol. Soc..

[19] Owsley C (2002). Timed instrumental activities of daily living tasks: Relationship to cognitive function and everyday performance assessments in older adults. Gerontology.

[20] Tucker-Drob EM (2011). Neurocognitive functions and everyday functions change together in old age. Neuropsychology.

[21] Bell-Mcginty S (2002). Standard measures of executive function in predicting instrumental activities of daily living in older adults. Int. J. Geriatr. Psychiatry.

[22] Cahn-Weiner DA, Boyle PA, Malloy PF (2002). Tests of executive function predict instrumental activities of daily living in community-dwelling older individuals. Appl. Neuropsychol..

[23] Nguyen CM (2020). Contribution of executive functioning to instrumental activities of daily living in older adults. Appl. Neuropsychol. Adult.

[24] Kail R, Salthouse TA (1994). Processing speed as a mental capacity. Acta Psychol. (Amst.).

[25] Harvey PD (2019). Domains of cognition and their assessment. Dialogues Clin. Neurosci..

[26] Glisky EL, Riddle DR (2007). Changes in cognitive function in human aging. Brain Aging: Models, Methods, and Mechanisms.

[27] Lindsay K, Bone I, Fuller G (2010). Clinical presentation, anatomical concepts and diagnostic approach. Neurol Neurosurg Illus.

[28] Diamond A (2013). Executive functions. Annu. Rev. Psychol..

[29] Nasreddine ZS (2005). The Montreal Cognitive Assessment, MoCA: A brief screening tool for mild cognitive impairment. J. Am. Geriatr. Soc..

[30] Gold DA (2012). An examination of instrumental activities of daily living assessment in older adults and mild cognitive impairment. J. Clin. Exp. Neuropsychol..

[31] Rajan KB (2013). Disability in basic and instrumental activities of daily living is associated with faster rate of decline in cognitive function of older adults. J. Gerontol. Ser. A Biol. Sci. Med. Sci..

[32] Sigrist AAF, Becattini-Oliveira AC, Charchat-Fichman H (2021). Patterns of instrumental activities of daily living between community-dwelling older adults. Dement. Neuropsychol..

[33] Boucą-Machado R, Maetzler W, Ferreira JJ (2018). What is functional mobility applied to Parkinson’s disease?. J. Parkinsons Dis..

[34] Forhan M, Gill SV (2013). Obesity, functional mobility and quality of life. Best Pract. Res. Clin. Endocrinol. Metab..

[35] Donoghue OA (2014). Using timed up and go and usual gait speed to predict incident disability in daily activities among community-dwelling adults aged 65 and older. Arch. Phys. Med. Rehabil..

[36] Shimada H (2010). Predictive validity of the classification schema for functional mobility tests in instrumental activities of daily living decline among older adults. Arch. Phys. Med. Rehabil..

[37] Herold F (2018). Thinking while moving or moving while thinking—Concepts of motor-cognitive training for cognitive performance enhancement. Front. Aging Neurosci..

[38] Karssemeijer EGA (2017). Positive effects of combined cognitive and physical exercise training on cognitive function in older adults with mild cognitive impairment or dementia: A meta-analysis. Ageing Res. Rev..

[39] Meng Q (2022). The effect of combined cognitive intervention and physical exercise on cognitive function in older adults with mild cognitive impairment: A meta-analysis of randomized controlled trials. Aging Clin. Exp. Res..

[40] Lam LCW (2015). Would older adults with mild cognitive impairment adhere to and benefit from a structured lifestyle activity intervention to enhance cognition?: A cluster randomized controlled trial. PLoS One.

[41] Jardim NYV (2021). Dual-task exercise to improve cognition and functional capacity of healthy older adults. Front. Aging Neurosci..

[42] Maffei L (2017). Randomized trial on the effects of a combined physical/cognitive training in aged MCI subjects: The Train the Brain study. Sci. Rep..

[43] Ball K, Edwards JD, Ross LA (2007). The impact of speed of processing training on cognitive and everyday functions. J. Gerontol. Ser. B Psychol. Sci. Soc. Sci..

[44] Edwards JD (2013). An examination of mediators of the transfer of cognitive speed of processing training to everyday functional performance. Psychol. Aging.

[45] Salthouse T (1996). The Processing-speed theory of adult age differences in cognition. Psychol. Rev..

[46] Demnitz N (2016). A systematic review and meta-analysis of cross-sectional studies examining the relationship between mobility and cognition in healthy older adults. Gait Posture..

[47] Bruijnen CJWH (2020). Psychometric properties of the Montreal Cognitive Assessment (MoCA) in healthy participants aged 18–70. Int. J. Psychiatry Clin. Pract..

[48] Tsai CF (2012). Psychometrics of the Montreal Cognitive Assessment (MoCA) and its subscales: Validation of the Taiwanese version of the MoCA and an item response theory analysis. Int. Psychogeriatr..

[49] Chen RH, Chen HY (2002). Chinese Version of Wechsler Adult Intelligence Scale Manual.

[50] Jaeger J (2018). Digit symbol substitution test: The case for sensitivity over specificity in neuropsychological testing. J. Clin. Psychopharmacol..

[51] Kuo HY, Hua MS (2015). Chinese version of Color Trails Test Manual.

[52] Hua MS (2005). Chinese Version of Wechsler Memory Scale Manual.

[53] Mathiowetz V (1985). Adult norms for the Box and Block Test of manual dexterity. Am. J. Occup. Ther..

[54] Podsiadlo D, Richardson S (1991). The Timed “Up & Go”: A test of basic functional mobility for frail elderly persons. Jags.

[55] Lawton MP, Brody EM (1969). Assessment of older people: Self-maintaining and instrumental activities of daily living. Gerontologist.

[56] Cohen RA, Marsiske MM, Smith GE (2019). Neuropsychology of Aging, Handb. Clin. Neurol..

[57] Gkotzamanis V (2021). Determinants of processing speed trajectories among middle aged or older adults, and their association with chronic illnesses: The English longitudinal study of aging. Life.

[58] Salthouse T (2012). Consequences of age-related cognitive declines. Annu. Rev. Psychol..

[59] Kuo HK (2007). Cognitive function, habitual gait speed, and late-life disability in the National Health and Nutrition Examination Survey (NHANES) 1999–2002. Gerontology.

[60] Makizako H (2015). Cognitive functioning and walking speed in older adults as predictors of limitations in self-reported instrumental activity of daily living: Prospective findings from the Obu Study of Health Promotion for the elderly. Int. J. Environ. Res. Public Health.

[61] Wadley V (2020). Cognitive processing speed is strongly related to driving skills, financial abilities, and other instrumental activities of daily living in persons with mild cognitive impairment and mild dementia. J. Gerontol. Med. Sci..

[62] Hausdorff JM (2005). Walking is more like catching than tapping: Gait in the elderly as a complex cognitive task. Exp. Brain Res..

[63] Bruce-Keller AJ (2012). Relationship between cognitive domains, physical performance, and gait in elderly and demented subjects. J. Alzheimer’s Dis..

[64] Demnitz N (2018). Cognition and mobility show a global association in middle- and late-adulthood: Analyses from the Canadian Longitudinal Study on Aging. Gait Posture.

[65] Morris R (2016). Gait and cognition: Mapping the global and discrete relationships in ageing and neurodegenerative disease. Neurosci. Biobehav. Rev..

[66] Rajtar-Zembaty A (2019). Global cognitive functioning and physical mobility in older adults with and without mild cognitive impairment: Evidence and implications. Folia Med. Cracov..

[67] Toots ATM (2019). Associations between gait speed and cognitive domains in older people with cognitive impairment. J. Alzheimer’s Dis..

[68] Artaud F (2015). Decline in fast gait speed as a predictor of disability in older adults. J. Am. Geriatr. Soc..

